# Serum biochemistry and haematology in wild and captive bearded seals (*Erignathus barbatus*) from Svalbard, Norway

**DOI:** 10.1186/s13028-021-00598-8

**Published:** 2021-08-26

**Authors:** Morten Tryland, Christian Lydersen, Kit Maureen Kovacs, Espen Rafter, Stein Istre Thoresen

**Affiliations:** 1 Department of Arctic and Marine Biology, UiT The Arctic University of Norway, Framstredet 39, 9037 Tromsø, Norway; 2 Department of Forestry and Wildlife Management, Inland Norway University of Applied Sciences, 2480 Koppang, Norway; 3 Norwegian Polar Institute, Fram Centre, 9296 Tromsø, Norway; 4 Polaria, Hjalmar Johansens gate 12, 2007 Tromsø, Norway; 5 Faculty of Veterinary Medicine, Norwegian University of Life Sciences, 1432 Ås, Norway

**Keywords:** Arctic, Clinical pathology, Environmental monitoring, *Erignathus barbatus*, Haematology, Marine mammal, Pinniped, Seal, Wildlife

## Abstract

**Background:**

Health assessment of seals in captivity include haematology and serum biochemistry measurements. Because such parameters differ between species, it is crucial to have species-specific reference values for the interpretation of clinical samples. Furthermore, differences in nutrition and environment, life cycles as well as seasonal/annual cycles and varying physiological conditions can potentially affect serum chemistry and haematology parameters. Blood samples from four captive adult bearded seals (initially caught as pups in Svalbard, Norway, now held at Polaria, an Arctic experience centre in Tromsø, Norway) collected over a 16-month period were analysed for haematology (n = 22) and serum chemistry (n = 25) parameters. Serum chemistry analyses were also conducted on blood samples from 74 wild bearded seals (1995–2007) collected from Svalbard, Norway.

**Results:**

We found higher activity of creatine kinase (CK) and higher concentrations of cortisol in the wild animals when compared to the captive seals, probably reflecting the physical restraint and concomitant stress induced during sampling. For the captive bearded seals, we did not find marked differences in haematology or serum chemistry parameters throughout the different seasons of sampling.

**Conclusions:**

This study presents haematology and serum chemistry reference values for captive and wild bearded seals. Comparing physiological parameters for captive seals with wild seals indicated that having wild-caught bearded seals under the conditions offered at Polaria for several years did not markedly affect physiological parameters of the animals, and that training may have helped to alleviate stress associated with blood sampling and veterinary inspection.

## Background

The bearded seal (*Erignathus barbatus*) is the largest northern phocid seal. This species is distributed, at low densities, throughout the circumpolar Arctic. Adults are 2–2.5 m long and weigh 250–300 kg or more. However, their weight varies quite dramatically on an annual cycle, and females, for example, have been recorded weighing more than 425 kg in spring [1]. The pups are born on ice floes during the spring, with peak pupping occurring in early May [2]. At birth pups are approximately 1.3 m long and weigh about 37 kg [2]. Pups enter the water shortly after being born and can swim and dive down to 200 m depth prior to weaning; older animals can dive deeper but rarely do because they feed in shallow, shelf waters. The pups stay close to their mothers for 2–3 weeks during the lactation period and weigh around 100 kg at weaning. Preferred bearded seal habitat is drifting pack ice in shallow areas over coastal shelves. Bearded seals are found at low densities in the fjords of Svalbard, Norway. They are predominantly benthic feeders, eating clams, shrimp, squid, crabs and fish. A study on stomach contents of bearded seals on Svalbard (n = 47) revealed a varied diet, with no significant differences between males and females. Polar cod (*Boreogadus saida*) was an important prey species that was found in 49% of the animals, proportionally in terms of frequency of occurrence followed by sculpins (*Cottidae* spp.; 44%), long rough dab (*Hippoglossoides platessoides*; 28%) and other fish species. In addition, molluscs (e.g., *Buccinum* spp.; 18%) and several other species of invertebrates were found in the stomachs [3]. Bearded seals have a longevity of 20–25 years in the wild [1].

Polaria is an Arctic experience centre located in Tromsø, Norway, which hosts aquariums with a variety of Arctic species including bearded seals. Seals in captivity are usually housed in an environment that is very different from the one in which they live in the wild. Captive environments often include differences in latitude (light conditions) and climatic conditions, feeding and nutrition, as well as variable possibilities and motivation for physical exercise. All of these parameters are important for the mental and physical health and well-being of an animal. Therefore, animals in captivity are often engaged in training programs to promote physical exercise and mental stimulation. These training programs also offer possibilities to get animals used to handling and to conduct clinical investigations and sampling without the need for anaesthesia. At Polaria, the bearded seals are trained to allow weighing and body condition assessment (palpation), inspection of mucosal membranes (eyes, mouth, nostrils, anus and reproductive organs), inspection of the flippers and the skin as well as biological sampling, such as blood sampling.

Without a proper training program, the possibilities for conducting a thorough clinical examination of captive bearded seals without the use of drugs or extreme restraint, would be impossible. Even if these samples were obtained, the lack of reference values for this species limits clinical interpretation. Therefore, obtaining and reporting haematology and serum chemistry parameters from bearded seals is extremely valuable. The aim of this study was to establish species-specific haematology and serum chemistry reference values for captive adult bearded seals and to provide comparative serum chemistry reference values from wild bearded seals.

## Methods

### Animals

Four bearded seals housed at Polaria, Tromsø, Norway were included in this study. These animals were caught as large pups in Kongsfjorden, Svalbard (78.9° N, 12.3° E) in 1998 (one male) and 2003 (three females). The seal pool at Polaria is approximately 100 m^2^ in size with a maximum depth of 4 m. The seals have access to the pool and “land” areas ad libitum. The indoor environment at Polaria is different from the High Arctic environment at Svalbard. However, the facility tries to replicate natural conditions as closely as possible, using, for example, a light regime that mimics the winter and summer season at higher latitudes. The water for the pool is obtained from the marine fjord just outside the facility at 20 m depth. The water temperature is at a minimum in February (1–2 °C) and at a maximum in August (< 10 °C) with some natural annual variation. The animals in this study were fed mainly Norwegian spring-spawning herring (*Clupea harengus*), sometimes mixed with capelin (*Mallotus villosus*). The animals were usually fed three times a day, as part of the training regime, as well as the performance and information dissemination programme with visitors to the facility. At the time of blood sampling the males were 8–9 years old and the females were 4–5 years old. A total of 25 blood samples were obtained during different months (February, March, April, May, June, August, November and December) throughout 2006–2008. Blood was sampled without previous fasting but before feeding. Blood was drawn from the plantar venous plexus of the hind flippers using blood collection tubes (full blood tubes for serum and EDTA tubes for plasma; Venoject®, Terumo, Leuven, Belgium) and 0.8 × 40 mm needles (Venoject®). Serum was prepared by centrifugation at 3000×*g* for 15 min and kept refrigerated. Full blood and serum were sent for analyses on the day of sampling and analysed the following day (Central Veterinary Laboratory, Norwegian University of Life Sciences) for haematology and serum biochemistry parameters (Table 1).

**Table 1:** Analytical methods used for serum biochemistry analyses of bearded seals (*Erignathus barbatus*) from Svalbard, Norway

Analyte^a^	Method/principle	Manufacturer^b^	References
AST	Modified IFCC^c^	Siemens	[4]
ALT	Modified IFCC	Siemens	[4]
ALP	Modified IFCC	Siemens	[4]
CK	*N*-acetylcysteine act., DGKC	Siemens	[4]
LDH	Enzymatic/Tris/NAD	Siemens	[4]
Amylase	Ethylidene blocked-pNPG7	Siemens	[4]
Lipase	Kinetic colorimetric	Sera-Pak	[5]
Albumin	Serum protein electrophoresis	Beckman	—
Bilirubin (total)	Diazo/caffeine	Siemens	[4]
Calcium	CPC	Siemens	[6]
Chloride	Ion-selective electrode, diluted	Siemens	[4]
Cholesterol	Enzymatic	Siemens	[7]
Cortisol	Solid-phase, chemiluminescent, competitive immunoassay	DPC	[8]
Creatinine	Jaffe, alkaline picrate, kinetic	Siemens	[9]
NEFA	ACS-ACOD-MEHA	Wako	[10]
Glucose	Hexokinase	Siemens	[11]
Magnesium	Xylidyl blue	Siemens	[12]
Phosphorus (inorg.)	Phosphomolybdate/ultraviolet	Siemens	[13]
Potassium	Ion-selective electrode, diluted	Siemens	[4]
Protein (total)	Biuret	Siemens	[4]
Sodium	Ion-selective electrode, diluted	Siemens	[4]
Thyroxine	Solid-phase, chemiluminescent, competitive immunoassay	Siemens	[14]
Triglycerides	GPO, trinder without serum blank	Siemens	[15]
Urea	Urease with GLDH	Siemens	[16]

^a^*AST* Aspartate aminotransferase, *ALT* Alanine aminotransferase, *ALP* Alkaline phosphatase, *CK* Creatine kinase, *LDH* Lactate dehydrogenase, *NEFA* Non-esterified fatty acids

^b^Siemens Healthcare Diagnostics (including Sera-Pak), Tarrytown, NY, USA; Beckman Coulter, Inc, Fullerton, CA, USA; DPC: Diagnostic Products Corporation, Los Angeles, CA, USA; Wako Chemicals GmbH, Neuss, Germany

^c^*IFCC* International Federation of Clinical Chemistry, *pNPG7* *p*-nitro-phenylmalto-heptaoside, *NAC* *N*-acetylcysteine, *DGKC* Deutsche Gesellschaft für Klinische Chemie, *CPC* *o*-cresolphtalein complexone, *ACS-ACOD-MEHA* Acyl-CoA-Synthetase-Ascorbate oxidase- 3-Methyl-*N*-Ethyl-*N*-(ß-hydroxyethyl)aniline, *GPO* glycerol-3-phosphate oxidase, *GLDH* glutamate dehydrogenase

Wild bearded seals (n = 74) were sampled in Kongsfjorden, Svalbard (78.9° N, 12.3° E) in 1995 (n = 13), 2005 (n = 10), 2006 (n = 17) and 2007 (n = 34). All animals were caught and sampled in the month of May while the pups were still nursing. For these procedures, the animals were physically restrained without using anaesthesia or sedation. The sampled animals included seven adults (all females) and 67 pups (22 females, 45 males). The average weight of the pups was 53.8 kg (range 27–101 kg) and the average weight of adults was 339 kg (range 237–421 kg). Blood was obtained from the extradural intra-vertebral vein using either a 2.1 × 80 mm or 2.1 × 150 mm needle (depending on the age of the animal) (Sterican®, VWR International, Oslo, Norway) mounted on a 50 mL syringe. The blood was transferred into blood collection tubes (full blood tubes for serum preparation; Venoject®) and serum was prepared through centrifugation (as described above). The samples from the wild bearded seals were then stored at − 20 °C until analyses were undertaken in 2008 (Central Veterinary Laboratory, Norwegian University of Life Sciences). Due to the unavoidable delay from sampling of wild animals to analysis, haematological analysis was not possible for samples from wild bearded seals as haematological parameters are not stable and should be analyzed within 24 h.

### Haematology analyses

Haematology parameters were assayed for 22 samples (from the four captive individuals) with an ADVIA® 2120 Hematology System using ADVIA 2120 MultiSpecies™ System Software (Siemens Healthcare Diagnostics, Tarrytown, NY, USA). The reference values for haematology parameters were calculated in Excel and are presented as mean, due to a restricted number of measurements, and standard deviation to indicate the variation.

### Serum chemistry and endocrinology analysis

A total of 25 serum samples from four captive bearded seals and serum samples from 74 wild bearded seals were analysed for selected enzymes, proteins, metabolites, minerals, cortisol, and thyroxine (Table 1). Serum chemistry analyses were assayed using an ADVIA® 1650 System (Siemens Healthcare Diagnostics, USA). Lipaemia was indicated by the ADVIA 1650 System, and samples classified as lipaemic (1+ and 2+) were automatically diluted before analysis to avoid erroneous measurements [17]. Serum endocrinology analyses (cortisol and thyroxine) were assayed using an IMMULITE® 2000 Immunoassay System (Siemens Healthcare Diagnostics, USA). The reference values for serum biochemistry parameters and hormones were calculated in Excel and are presented as median, range (min–max) and percentiles (5–95%), to show variation and a reference range that is excluding outliers.

## Results

Haematology results (captive seals only) are presented in Table 2 for each individual (n = 4) and for all samples (n = 22) combined. Serum biochemistry parameters for wild bearded seals (pups and adults) and captive adult bearded seals are presented in Table 3.

**Table 2:** Haematology parameters for four captive bearded seals (*Erignathus barbatus*) over a period of 19 months (2006–2008), presented as mean (standard deviation) for each individual and combined

Parameter^a^	Individual seals^b^ (number of samples from each seal)				All samples combined (n = 22)
	”Diesel” ♂ (n = 2)	”Aurora”♀ (n = 6)	”Maisan” ♀ (n = 7)	”Bella” ♀ (n = 7)	
WBC (10^9^/L)	9.5	11.6 (2.4)	7.4 (0.8)	9.5 (2.6)	9.5 (1.2)
RBC (10^12^/L)	3.8	4.0 (0.3)	3.7 (0.1)	3.8 (0.2)	3.8 (0.1)
HGB (g/L)	217	211 (24)	231 (12)	230 (16)	217 (20)
HCT (L/L)	0.45	0.51 (0.04)	0.54 (0.02)	0.54 (0.02)	0.50 (0.10)
MCV (fL)	122	128 (15)	146 (5)	142 (7)	135 (13)
MCHC (g/L)	435	414 (28)	429 (11)	430 (31)	427 (3)
RDW (%)	16.8	16.8 (4.7)	12 (0.7)	13.5 (2.1)	14.8 (2.5)
PLT (10^9^/L)	220	305 (59)	224 (132)	216 (76)	241 (4)
Neutrophils (10^9^/L)	5.1	6.7 (1.2)	4.7 (0.5)	5.6 (0.4)	5.5 (0.4)
Lymphocytes (10^9^/L)	2.5	2.6 (0.6)	1.6 (0.3)	2.5 (2.3)	2.3 (0.5)
Monocytes (10^9^/L)	0.9	1.2 (0.4)	0.7 (0.1)	1.0 (0.3)	1.0 (0.1)
Eosinophils (10^9^/L)	0.8	1.0 (0.6)	0.3 (0.2)	0.3 (0.2)	0.6 (0.3)
Basophils (10^9^/L)	0.3	0.1 (0.1)	0.0 (0.0)	0.1 (0.1)	0.1 (0.1)

^a^*WBC* white blood cell count, *RBC* red blood cell count, *HGB* hemoglobin, *HCT* hematocrit, *MCV* mean corpuscular volume, *MCHC* mean corpuscular hemoglobin concentration, *RDW* red cell distribution width, *PLT* platelet count

^b^“Diesel” was 8–9 years old and “Aurora”, “Maisan” and “Bella” were 4–5 years old at the time of sampling. “Diesel” was sampled only twice and results are presented as mean, not including standard deviation

**Table 3:** Clinical serum biochemistry parameters for wild (Svalbard, Norway) and captive bearded seals (*Erignathus barbatus*), presented as median, range and 5–95 percentiles

Analyte^a^	Pups (wild)				Adults (wild)				Adults (captive)			
	n^b^	Median	Range	5–95%	N	Median	Range	5–95%	N	Median	Range	5–95%
AST (U/L)	67	79	49–198	53–148	7	46	39–106	39–96	25	58	35–89	37–84
ALT (U/L)	67	8	0–24	1–16.4	7	5	1–33	1.3–27.6	25	41	28–60	33–59
ALP (U/L)	67	556	232–961	299–868	7	56	14–110	15–102	25	123	6–260	27–196
CK (U/L)	67	385	114–3383	136–2346	7	126	55–1234	56–1147	25	150	89–458	97–286
LDH (U/L)	67	669	271–1897	329–1175	7	185	89–1245	97–1028	25	354	201–965	254–812
Amylase (U/L)	67	1	0–9	0–5	7	1	0–6	0.3–4.8	23	1	0–4	0–2
Lipase (U/L)	67	25	11–54	14–47	7	142	52–156	62–156	25	241	69–331	124–297
Protein, total (g/L)	67	59	49–69	53–65	7	69	64–83	64–80	25	63	55–75	57–72
Albumin (g/L)	34	40.5	31.0–48.6	32.6–44.2	5	36.5	35.6–41.9	35.6–41.5	25	35	29–38	32–38
Globulin total (g/L)	34	19	14–26	15–22	5	33	28–41	29–40	25	29	25–39	25–34
α1-globulins (g/L)	34	3.9	2.9–5.4	3.2–4.9	5	4.4	4.3–5.1	4.3–5.0	25	5	3–9	3.3–8.5
α2-globulins (g/L)	34	4.7	3.2–7.4	3.5–6.7	5	7.5	6.6–7.8	6.6–7.8	25	10	6–13	6.7–12.9
β-globulins (β1 + β2; g/L)	34	7.9	5.9–11.8	6.1–9.5	5	13.8	10.4–16.4	10.4–16.7	25	8	6–13	6.7–10.9
γ-globulins (g/L)	34	2	0.6–4.8	0.7–4.4	5	7.5	6.7–12.5	6.8–12.1	25	6	3–9	3.2–8.2
Total bilirubin (µmol/L)	67	1	0–11	0–7.4	7	1	1–4	1–3.4	25	1	0–6	1–3.6
Urea (mmol/L)	67	9.8	4.5–19.0	6.6–16.2	7	8.2	6.3–9.3	6.4–9.1	25	6	4–10	4.5–8.8
Creatinine (µmol/L)	67	119	77–362	92–180	7	173	153–213	155–209	25	139	110–194	118–158
Cholesterol (mmol/L)	67	6	1.9–10	3.0–9.0	7	3	2.6–5.7	2.6–5.4	25	5	4–7	3.9–6.9
NEFA (mmol/L)	67	1.2	0.3–3.2	0.5–2.3	7	1	0.3–1.4	0.5–1.4	25	0	0–1	0.02–0.56
Glucose (mmol/L)	67	6.8	1–13.4	3.3–10.6	7	6.6	1.2–12.2	2.5–11.1	25	6	3–8	3.1–7.2
Calcium (mmol/L)	67	2.6	1.1–3.0	2.4–2.9	7	1.9	1.2–2.5	1.3–2.5	25	2	2–3	2.1–2.5
Phosphate (inorg.; mmol/L)	67	3	1.5- 5.6	2.0–4.1	7	2.3	0.7–3.6	0.9–3.5	25	2	2–3	1.5–2.3
Magnesium (mmol/L)	67	1.1	0.7–2.1	0.9–1.7	7	1	0.8–1.3	0.8–1.3	25	1	1–1	0.9–1.2
Sodium (mmol/L)	67	153	131–159	146–157	7	153	149–166	149–164	23	153	150–160	151–157
Potassium (mmol/L)	67	4.1	3.1–5.9	3.5–4.6	7	4.1	3.7–4.7	3.7–4.6	23	4	4–5	3.7–4.9
Chloride (mmol/L)	67	102	86–107	97–107	7	106	102–108	103–108	23	114	107–117	110–116
Total T4 (nmol/L)	67	49	11–151	23–118	7	25	13–29	14.2–28.1	21	23	9–74	10–62
Cortisol (nmol/L)	67	398	199–977	212–832	7	1010	612–1206	674–1162	21	345	236–549	265–491

^a^*AST* Aspartate aminotransferase, *ALT* Alanine aminotransferase, *ALP* Alkaline phosphatase, *CK* Creatine kinase, *LDH* Lactate dehydrogenase, *NEFA* Non-esterified fatty acids

^b^n indicates the number of animals that were sampled and analyzed for each specific analyte. For captive adult bearded seals, the data is based on blood samples obtained from four 9–10-year-old bearded seals sampled 2–7 times each (2006–2008)

From the wild bearded seals, 36 samples were classified by the ADVIA system as being slightly lipaemic (1+) and 28 were classified as moderately lipaemic (2+). When comparing results from samples with no or slight lipaemia (1+) to samples with moderate lipaemia (2+), we found no obvious differences for parameters previously reported for marine mammal sera that might potentially be affected by moderate lipaemia, such as creatine kinase (CK), alkaline phosphatase (ALP), lactate dehydrogenase (LDH), lipase, urea, creatinine, sodium and chloride [18]. No lipaemia was recorded for the serum samples obtained from the captive bearded seals.

Serum activity of CK was higher in wild pups (mean 622 U/L, *SD* 684, range 114–3383 U/L) than in wild adults (mean 389 U/L, *SD* 488, range 55–1239 U/L) and captive adults (mean 172 U/L, *SD* 84, range 89–458 U/L). Cortisol concentration was higher in wild adults (mean 956 nmol/L, *SD* 193, range 612–1206 nmol/L) than in wild pups (mean 437 nmol/L, *SD* 189, range 199–977 nmol/L) or in captive adults (mean 352 nmol/L, *SD* 74, range 236–549 nmol/L).

## Discussion

We present herein the first thorough set of haematology and serum biochemistry reference values for captive (adult) bearded seals and serum biochemistry reference values for wild (adult and pup) bearded seals. These reference values may be valuable tools for assessing health status for this species, especially for bearded seals in captivity.

Haematology data on bearded seals is in general very limited; however, two studies report haematology parameters from wild bearded seals from the Chukchi Sea and Bering Sea [19, 20]. Compared to the values reported in these studies, the values for haematocrit (HCT), haemoglobin (Hb/HGB), and mean corpuscular haemoglobin concentration (MCHC) for the four captive adult bearded seals in our study were within the same range. It should be noted, in any case, that the studies of the Chukchi Sea and Bering Sea bearded seals were, similar to our study, based on only a few individuals (one adult, six subadults and one pup).

The total globulin concentrations were higher in wild adult bearded seals compared to wild pups, and higher in adult wild bearded seals compared to captive adults. In particular, the concentrations of the gamma globulins were higher in wild adults compared to both captive adults and wild pups. The reasons for this are not obvious, but it could be hypothesised that the immune system of wild adults might be exposed to more challenges, provoking infections and inflammation resulting in increased serum concentrations of gamma globulins. The lower gamma globulin levels in pups might reflect their immature immune system.

From the serum biochemistry analyses, it was clear that the ALP activity was higher in the serum from wild pups, as compared to both wild and captive adult seals. This is in line with findings from White Sea bearded seals (*E. b. barbatus*). Animals that were 3–5 years old had higher levels of ALP compared to animals that were ten years of age [21]. The limited sample size in that (and our) study limits interpretation of these results, although higher ALP activity in younger individuals is a well-known finding in other seal species (e.g., hooded seals; *Cystophora cristata*) and harp seals (*Phoca groenlandica*) [22], as well as in humans [23]. As the pups in our study were quite young, a few days to 2–3 weeks, ALP results should be interpreted with these age effects in mind.

The serum CK activity was highest in wild pups, followed by wild adults and lowest in captive adults. Creatine kinase is found in tissues that consume adenosine-triphosphate (ATP) rapidly, such as muscle tissue. Creatine kinase activity in serum is used as a marker of muscle disease or trauma [24–26]. In addition, in several seal species, higher concentrations of this enzyme have been found in muscle tissue compared to other body tissues [24], and high CK activity in serum has been associated with handling stress in harbor seals (*Phoca vitulina*) [27]. Thus, the higher CK activity found in wild bearded seal adults and pups, compared to captive individuals, may reflect the stress and physical activity associated with netting and restraint of the animals during sampling. This suggestion is also supported by the serum cortisol levels, which were highest in wild adults, followed by wild pups, and lowest in captive animals. Glucocorticoids (corticosterone and cortisol) are mediators of the physiological stress response in mammals. Although cortisol in blood serum has been extensively used as a stress marker, care should be taken when interpreting its values. Cortisol is released in response to a stressor almost immediately, causing spikes in the blood cortisol concentrations; therefore, one should be aware that, particularly in wild animals, cortisol concentrations may be affected by the stress induced even by the blood sampling itself [28]. Still, it is notable that in our study both the CK levels and cortisol levels were lower for the captive bearded seals. Although this comparison is based on few individuals, these results support the benefit of training captive animals to cooperate during veterinary investigations and sampling to reduce stress responses.

Seasonal variations of several physiological parameters may be observed in wild populations due to seasonal changes in environmental conditions. However, we did not find any indications of seasonal differences for the captive bearded seals of this study; suggesting that these animals might have a steady physiological state throughout the year.

## Conclusions

The results of this study represent haematology and serum chemistry reference values for captive bearded seals and serum chemistry reference values for wild bearded seals. The values for both groups were similar for most parameters suggesting that holding wild-caught bearded seals at Polaria for several years has not altered the animals’ serum chemistry parameters substantially from what is normal for wild animals. Furthermore, it appears that Polaria’s training regime has largely alleviated stress associated with blood sampling and general veterinary inspection.

## Data Availability

The datasets used and/or analyzed during the current study are available from the corresponding author upon request.
