# Correction to: Serum biochemistry and haematology in wild and captive bearded seals (*Erignathus barbatus*) from Svalbard, Norway

**DOI:** 10.1186/s13028-021-00602-1

**Published:** 2021-09-08

**Authors:** Morten Tryland, Christian Lydersen, Kit Maureen Kovacs, Espen Rafter, Stein Istre Thoresen

**Affiliations:** 1 Department of Arctic and Marine Biology, UiT The Arctic University of Norway, Framstredet 39, 9037 Tromsø, Norway; 2 Department of Forestry and Wildlife Management, Inland Norway University of Applied Sciences, 2480 Koppang, Norway; 3 Norwegian Polar Institute, Fram Centre, 9296 Tromsø, Norway; 4 Polaria, Hjalmar Johansens gate 12, 2007 Tromsø, Norway; 5 Faculty of Veterinary Medicine, Norwegian University of Life Sciences, 1432 Ås, Norway

## Correction to: Acta Vet Scand (2021) 63:33 10.1186/s13028-021-00598-8

Following the publication of the original article [1], we were notified of erroneously presented units for red and white blood cells in Table 2 and missing unit for Total T4 in Table 3.

Corrected units are represents in Bold:

**Table 2**

WBC (**10**^**9**^/L)

RBC (**10**^**12**^/L)

HGB (g/L)

HCT (L/L)

MCV (fL)

MCHC (g/L)

RDW (%)

PLT (**10**^**9**^/L)

Neutrophils (**10**^**9**^/L)

Lymphocytes (**10**^**9**^/L)

Monocytes (**10**^**9**^/L)

Eosinophils (**10**^**9**^/L)

Basophils (**10**^**9**^/L)

**Table 3**

Total T4 **(nmol/L)**
